# Vincent's Angina

**Published:** 1910-12-31

**Authors:** 


### VINCENT'S ANGINA.

Vincent's angina is a faucial lesion, usually unilateral, characterised by deep ulceration of the tonsil and adjacent structures, a peculiar fœtor, and enlargement of the corresponding lymph glands. Etiologically, it is associated with the symbiosis in the local lesion of two organisms—a fusiform bacillus and a spirillum, described by Vincent in 1896. Similar organisms have been found in cases of hospital gangrene.

### Diagnosis.

The importance of the condition lies in the fact that it may be mistaken for diphtheria, syphilis, or for malignant ulceration of the tonsil. It is diagnosed by the detection of the bacillus fusiformis and the spirillum together in films prepared from swabbing in the same way as in the immediate detection of diphtheria. Dr. J. D. Rolleston has written a capital article upon the subject in the Annual Report of the Metropolitan Asylums Board for 1909; and although he allows that it is very much rarer than is diphtheria, he points out that since he first became familiar with this form of sore throat he realises that he had previously seen several cases which, on retrospection, were probably examples of this condition, but which he had not learned to recognise as such. Many other observers have doubtless had a similar experience. From statistics based upon cases sent to isolation hospitals as diphtheria it is certain that Vincent's angina constitutes at least 0.5 per cent. of all forms of sore throat and 3.1 per cent. of cases of non-diphtheritic angina. These figures probably underestimate its real frequency. The more carefully it is sought for, the more often is it found to be the correct diagnosis in cases of sore throat that may at first be regarded as of some other kind.

It is far more common in children than in adults; males and females are affected in equal proportions.

### Contagiousness.

The disease is not very contagious, at any rate amongst adults. During five years of careful observation upon this point at the Grove Hospital, none of the staff contracted the disease, though they were subject to various forms of sore throat, including 196 cases of follicular tonsillitis and nineteen of quinsy; whilst their susceptibility to infection was further shown by the fact that forty developed scarlet fever and thirty-seven diphtheria during the five years. Nevertheless, Vincent's angina, as might be expected from its bacteriology, is catching. Small epidemics have been reported, especially in children's homes. The disease has been conveyed by kissing or by the use of an infected pipe or glass, as Vincent himself showed. A dentist has been infected by his patient, and *vice versa*. Family epidemics in which father, mother, and children were all affected are on record. Buhlig describes an outbreak amongst medical students who used a tobacco-pouch in common, the string of which they fastened with their teeth. Todd states that a pathologist caught the disease himself after examining throats during an epidemic of Vincent's angina in a lunatic asylum.

### Oral Sepsis.

Some writers have laid special stress on general ill-health and oral sepsis as predisposing causes, but others find the disease equally frequent amongst those who have previously been perfectly well. Carious teeth are present in most cases, but not to a greater extent than in most children of the hospital class upon whom statistics are mainly based.

There is no particular seasonal incidence, unless perhaps that the disease is a little more prevalent in the warmer than it is in the colder months.

### Clinical Types.

It is customary to distinguish two main forms of Vincent's angina—an ulcerative and a membranous or diphtheroid; Rolleston holds, however, that the former is but a more advanced stage of the latter. The slough which covers the ulcer may simulate diphtheritic membrane so closely that even after considerable experience the condition may be regarded as diphtheria and treated accordingly, especially as the characteristic fœtor is often absent in the earlier stage. No help in diagnosis can be gained from the history of the onset, for the prodromal symptoms are merely those that are common to any anginal sore throat. Soreness of the throat is an almost constant symptom, and next in frequency come swelling of the glands in the neck, headache, vomiting, and shivering. Nasal discharge also occurs in many cases, just as it so often does at the beginning of diphtheria.

The resemblance to severe diphtheria is apt to be increased by the presence of faucial œdema. Though cervical adenitis may be considerable, it is seldom, if ever, suppurative.

The fœtor of Vincent's angina, though absolutely characteristic and quite distinct from that of malignant diphtheria, may mislead those who have had no experience of the former disease.

It is a unilateral affection in the great majority of cases, or, if both sides of the fauces are involved, the lesions are predominant on one side. The uvula is apt to be affected also, and it has even been completely destroyed by the ulceration, though damage to it is generally less considerable, and regeneration of tissue occurs. The larynx is seldom involved, but ulcero-membranous stomatitis due to the same fuso-spirillar symbiosis has been recorded both in association with and independently of the faucial lesion.

### The Temperature.

Disproportion between the severity of the local and general symptoms is one of the most striking features of Vincent's angina. The constitutional disturbance is generally slight, or at least lasts only during the pyrexial period, which in most cases is short. In five of Rolleston's thirty-two cases the temperature was normal throughout the period of observation, though the local process was still in an acute stage on admission; in ten it ranged between 99° and 100° F.; in only four did it rise above 102° F., the highest reading being 103.8° F. In eleven cases the temperature became normal within twenty-four hours of admission, and in only two did the pyrexia last for more than four days after their arrival.

### Duration.

Compared with diphtheria, however, the specific disease which it most closely resembles, Vincent's angina runs a protracted course. A diphtheritic throat generally becomes clean within a few days of the injection of antitoxin; the healing process in Vincent's angina requires a much longer period as a rule. In Rolleston's series of thirty-two the average course was eighteen days, the extreme limits being five days in the mildest and fifty-nine days in the most severe. A still more chronic course has been recorded by several writers. Bayer had a case which lasted between three and four months, defying all local treatment and recovering ultimately under strengthening diet and arsenic. Pusateri's case lasted for over a year, tuberculous ulceration of the tonsils having been at one time feared.

Relapses occurred in two of Rolleston's cases. One occurred on the ninth day, and was attributed to accidental inoculation during painting of the throat as the child struggled. The other relapse occurred without obvious cause on the twenty-fourth day. In both cases the right tonsil and right side of the uvula were involved in the relapse, whereas the left tonsil and left side of the uvula had been affected in the initial attack.

As a rule the fuso-spirillar organisms disappear as healing commences, the fusiform bacilli persisting longer than the spirilla.

### Complications.

Fortunately Vincent's angina is seldom accompanied or followed by complications of any serious nature. The cervical glands do not suppurate; otitis media does not ensue, nor does paralysis. There may be transient albuminuria in a few cases. It is said that when stomatitis accompanies the faucial lesion septic sequelæ are more liable to occur, but luckily the general stomatitic form is itself exceptional. A small number of cases in which death has resulted from septic broncho-pneumonia have been recorded, but upon the whole, severe though the local lesion may appear to be, it has a very good prognosis.

### Treatment.

In most cases it is sufficient to swab the affected parts night and morning with undiluted tincture of iodine, as Vincent himself recommends. If fœtor is extreme, the throat may be syringed with a solution of potassium chlorate and myrrh, or with chlorine water. In several cases in which healing seemed to be delayed the application of powdered methylene blue to the ulcers has been followed by rapid improvement, and this remedy deserves further trial. The use of anti-diphtheritic serum seems to do no harm, so that if it has been given under the impression that the case was one of diphtheria the cure is not delayed; if anything, it is accelerated, so that the use of the serum in doubtful cases is not contraindicated. Internal medication is not essential; the needs of each particular case as regards malt and iron, arsenic, or the like will generally be obvious.

